# Chondroblastic Osteosarcoma of the Mandible in a Patient on Risedronate: A Rare Case of Neoadjuvant Chemotherapy Failure

**DOI:** 10.7759/cureus.19929

**Published:** 2021-11-26

**Authors:** Soufiane Boussouni, Gaoussou Touré

**Affiliations:** 1 Oral Surgery, Université de Paris, Paris, FRA; 2 Oral and Maxillofacial Surgery, Centre Hospitalier Intercommunal de Villeneuve Saint-Georges, Villeneuve Saint-Georges, FRA

**Keywords:** bone tumor, chemotherapy, mandible, jaw cancer, osteosarcoma, chondroblastoma

## Abstract

Osteosarcoma of the jaw only represents 0.5-1% of tumors of the facial mass. Due to its rarity, clinical diagnosis is thus difficult. The guidelines for this pathology remain unclear, and the need for neoadjuvant chemotherapy is still debated. This case report aims to describe a rare case of chondroblastic osteosarcoma in a 50-year-old woman on risedronate treated by neoadjuvant chemotherapy. The tumor extended from the mandibular left first premolar to the mandibular right canine. An excisional biopsy was performed, leading to a diagnosis of chondroblastic osteosarcoma. Neoadjuvant chemotherapy was ineffective, as it did not result in the shrinkage of the tumor. A pelvi-mandibulectomy with fibula free flap reconstruction of the mandible was subsequently successfully performed followed by radiotherapy.

## Introduction

Osteosarcoma is one of the most frequent malignant bone tumors [1,2]. However, only 5-10% of cases are localized in the jaws, corresponding to 0.5-1% of tumors of the facial mass [3]. Osteosarcoma of the jaw (OSJ) most commonly occurs in the third or fourth decade of life [3]. There is no association known between bisphosphonate and OSJ although some authors have shown that risedronate can inhibit human osteosarcoma cell invasion [4]. The diagnosis is difficult because of the varied histological and clinical findings. The differential diagnosis between chondroblastic osteosarcoma and chondrosarcoma is also challenging. Although the need for neoadjuvant chemotherapy has been well described since the 1980s in long bone osteosarcoma [5,6], its usage still remains debated for OSJ. Because of its rarity, the recommendations and standards of care are still controversial, with a lack of guidelines concerning this pathology. Indeed, despite the collaborative work of the European and American Osteosarcoma Study Group (EURAMOS-1), OSJ was excluded from the protocol. A large surgical resection is, however, almost always necessary. The aim of this article is to report and discuss the management of chondroblastic osteosarcoma in a 50-year-old woman undergoing neoadjuvant chemotherapy.

## Case presentation

**Figure 1:**
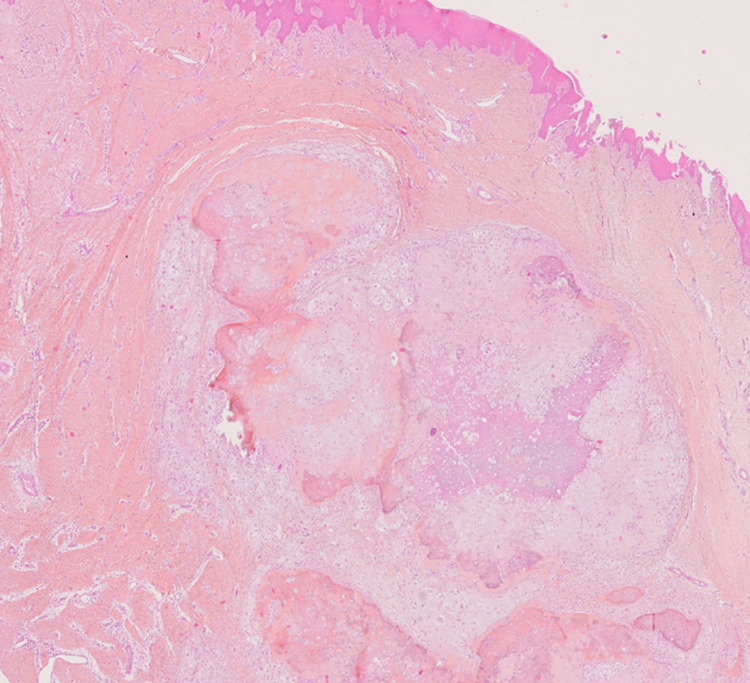
Photomicrograph (H&E x40) of the cartilaginous tissue. The chondroid matrix presents chondrocytes with cytonuclear abnormalities.

**Figure 2:**
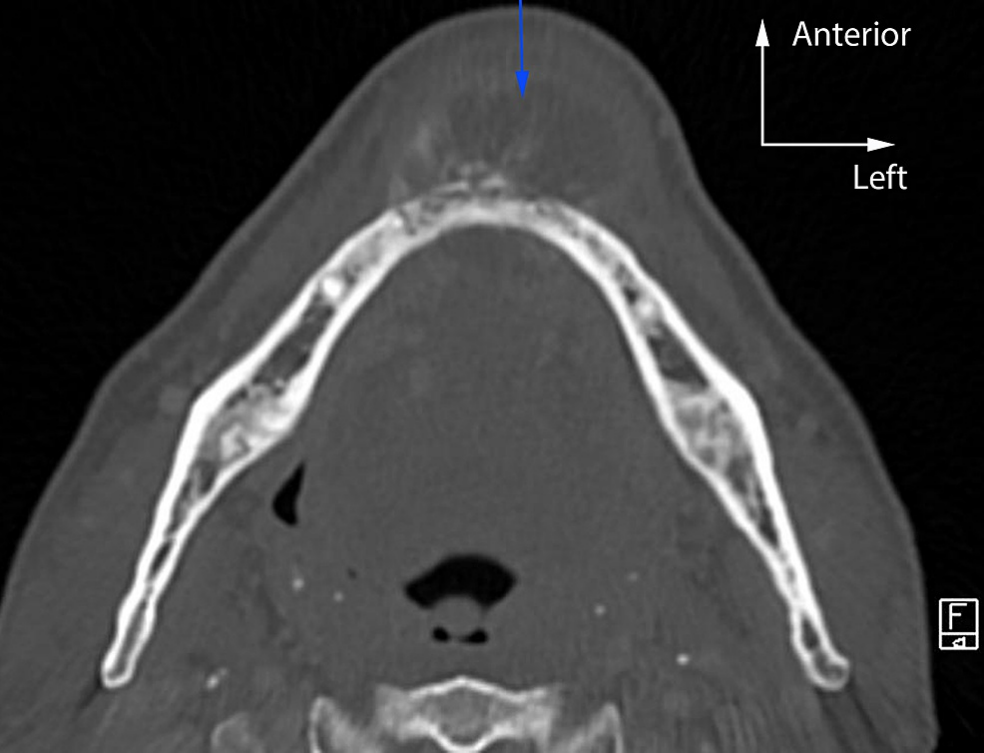
Computed tomography scan of the 38-mm-long axis lesion in the symphyseal region.

A 50-year-old woman was referred by her dentist for the evaluation of mandibular swelling. The patient had osteoporosis treated with risedronate. Physical examination revealed swelling of the mandibular symphysis with chin deformation. The tumor did not seem to adhere to the skin but extended from the mandibular left first premolar to the mandibular right canine. The anterior buccal floor was deformed, and the inferior alveolar nerve was preserved. An excisional biopsy of the buccal mass was performed, confirming an initial diagnosis of chondrosarcoma. The histological plates were reinterpreted, leading to a final diagnosis of chondroblastic osteosarcoma. Cartilaginous tissue with chondroid matrix was observed in which chondrocytes with cytonuclear abnormalities were found (Figure 1). Immunohistochemistry was not contributory, because it did not detect any IDH1 and IDH2 variants. Craniofacial computed tomography scan (Figure 2) revealed a 32-mm-long axis lesion in the symphyseal region responsible for an osteolysis on both sides of the mandible. This scan did not reveal any suspicious lesions elsewhere. No suspected hypermetabolic focus or submandibular and cervical lymph node involvement was found in positron emission tomography imaging.

It was decided to implement neoadjuvant chemotherapy with doxorubicin 80 mg and cisplatin 140 mg over three cycles. Due to the lack of significant evolution in the tumor mass, an interrupted pelvi-mandibulectomy with bilateral lymph node dissection was performed along with reconstruction with a fibula free flap. The histological analysis after the tumor resection showed a lobulated architecture of the tumor, with pleomorphic cells associated with a predominantly cartilaginous matrix and a matrix in the form of thick immature bone areas (Figure 1). The lymph nodes were free of malignant cells. The final diagnosis was conventional osteosarcoma with a chondroblastic predominance. The patient underwent 60-Gy adjuvant radiotherapy in 33 fractions. At the time of writing, the patient was still undergoing treatment and will be followed in the long term.

## Discussion

This patient presented a lesion that was initially mistaken for osteitis. The lack of progress following treatment prompted her dentist to refer her to hospital. The clinical diagnosis of OSJ can be difficult, as it differs from osteosarcoma of the long bones by its clinical and biological aspects [2,7]. The most common symptom (85-95% of cases) is swelling [7,8]. The radiographic characteristics of osteosarcoma or chondrosarcoma are often difficult to interpret. The lesion is osteolytic to the mandible in 31% of cases, osteoblastic in 46%, and mixed in 23% [8]. The histological appearance of OSJ is similar to that of long bone sarcomas. The production of osteoid [8] by malignant cells, even in small quantities, makes it possible to diagnose osteosarcoma. In most cases, the chondroblastic form predominates [9].

Mutations in IDH1 and IDH2 genes constitute strong biomarkers of chondrosarcomas and aid in the differential diagnosis with chondroblastic osteosarcomas [10]. Indeed, the absence of mutation of these genes was, in this case, an additional argument for the diagnosis of chondroblastic osteosarcoma.

Given her bisphosphonate treatment, the lesion of our patient could have been akin to osteonecrosis of the mandible. Indeed, one of the side effect of bisphosphonate is osteonecrosis of jaws [11]. However, there was a low probability that it was osteonecrosis because of the oral treatment. Osteonecrosis of jaws related to oral bisphosphonate occurs only in one patient in 100,000 patients [12]. Further, the radiological aspect did not correspond to the usual presentation of this type of pathology. Finally, there are few data concerning the association between bisphosphonate and particularly risedronate and OSJ except some in vitro studies that have shown that bisphosphonates inhibit proliferation in a wide variety of tumor cell types including osteosarcoma [13].

The difficulty in establishing guidelines for OSJ treatment is mainly due to its rarity, as it represents only 0.5-1% of tumors of the facial mass [1]. The consensus for osteosarcoma of the long bones is aggressive surgical resection with clean margins [9,10]. The National Comprehensive Cancer Network (NCCN) guidelines advocate neoadjuvant chemotherapy for all high-grade osteosarcomas of the long bones [14]. As OSJ can be low, medium, or high grade, neoadjuvant chemotherapy remains debated. According to some authors, neoadjuvant chemotherapy shrinks the tumor, which makes it easier to achieve negative margins [6]. Moreover, it provides surgeons with more preoperative time to plan the surgery and allows therapy to begin immediately instead of waiting for the initial surgery. In a retrospective review of 201 patients, Smeele et al. highlighted the statistically significant increase in survival if patients were treated with chemotherapy [6]. It would appear that neoadjuvant and/or adjuvant chemotherapy increases disease-free survival probabilities from 10-20 to 60% [15].

However, in the case of our patient, neoadjuvant chemotherapy did not shrink the tumor. There are very few data explaining the chemoresistance mechanism in osteosarcoma, and some authors evoke altered deoxyribonucleic acid (DNA) repair mechanism [16], drug inactivation [17], or altered cell cycle [18].

## Conclusions

This article reported a rare case of chondroblastic osteosarcoma in the mandible treated by neoadjuvant chemotherapy. The diagnosis of OSJ remains difficult from both a clinical and histopathological point of view. There are no guidelines regarding the use of chemotherapy and particularly the use of neoadjuvant chemotherapy which was unsuccessful in this case. This type of tumor can, nevertheless, be fatal. Clear standardized recommendations should, therefore, be put in place for the care of these patients.

## References

[1] Clark JL, Unni KK, Dahlin DC, Devine KD (1983). Osteosarcoma of the jaw. Cancer.

[2] Unni KK, Dahlin DC (1996). Dahlin’s Bone Tumors: General Aspects and Data on 11,087 Cases, 5th ed.

[3] Kassir RR, Rassekh CH, Kinsella JB, Segas J, Carrau RL, Hokanson JA (1997). Osteosarcoma of the head and neck: meta-analysis of nonrandomized studies. Laryngoscope.

[4] Xin ZF, Kim YK, Jung ST (2009). Risedronate inhibits human osteosarcoma cell invasion. J Exp Clin Cancer Res.

[5] Bramwell VH (1995). The role of chemotherapy in osteogenic sarcoma. Crit Rev Oncol Hematol.

[6] Smeele LE, Kostense PJ, van der Waal I, Snow GB (1997). Effect of chemotherapy on survival of craniofacial osteosarcoma: a systematic review of 201 patients. J Clin Oncol.

[7] Mardinger O, Givol N, Talmi YP, Taicher S (2001). Osteosarcoma of the jaw. The Chaim Sheba Medical Center experience. Oral Surg Oral Med Oral Pathol Oral Radiol Endod.

[8] Granowski-LeCornu M, Chuang SK, Kaban LB, August M (2011). Osteosarcoma of the jaws: factors influencing prognosis. J Oral Maxillofac Surg.

[9] Seng D, Wu J, Fang Q, Liu F (2019). Prognosis of osteosarcomas in the mandible: 15-year experience of 55 patients. Medicine (Baltimore).

[10] Kerr DA, Lopez HU, Deshpande V (2013). Molecular distinction of chondrosarcoma from chondroblastic osteosarcoma through IDH1/2 mutations. Am J Surg Pathol.

[11] Kennel KA, Drake MT (2009). Adverse effects of bisphosphonates: implications for osteoporosis management. Mayo Clin Proc.

[12] Khan AA, Morrison A, Hanley DA (2015). Diagnosis and management of osteonecrosis of the jaw: a systematic review and international consensus. J Bone Miner Res.

[13] Evdokiou A, Labrinidis A, Bouralexis S, Hay S, Findlay DM (2003). Induction of cell death of human osteogenic sarcoma cells by zoledronic acid resembles anoikis. Bone.

[14] Biermann JS, Chow W, Reed DR (2017). NCCN guidelines insights: bone cancer, version 2.2017. J Natl Compr Canc Netw.

[15] Thariat J, Julieron M, Brouchet A (2012). Osteosarcomas of the mandible: are they different from other tumor sites?. Crit Rev Oncol Hematol.

[16] Wang D, Luo M, Kelley MR (2004). Human apurinic endonuclease 1 (APE1) expression and prognostic significance in osteosarcoma: enhanced sensitivity of osteosarcoma to DNA damaging agents using silencing RNA APE1 expression inhibition. Mol Cancer Ther.

[17] Uozaki H, Horiuchi H, Ishida T, Iijima T, Imamura T, Machinami R (1997). Overexpression of resistance-related proteins (metallothioneins, glutathione-S-transferase pi, heat shock protein 27, and lung resistance-related protein) in osteosarcoma. Relationship with poor prognosis. Cancer.

[18] He H, Ni J, Huang J (2014). Molecular mechanisms of chemoresistance in osteosarcoma (Review). Oncol Lett.

